# A predictive model and mechanistic study of treatment effectiveness in patients newly diagnosed with small cell lung cancer

**DOI:** 10.3389/fonc.2025.1631490

**Published:** 2025-09-11

**Authors:** Tianyun Wang, Qiuyang Lu, Diexiao Luo, Chunfang Tao, Jiaqin Liu, Hongbo Zou, Qichao Xie, Rui Kong

**Affiliations:** Department of Oncology, The Third Affiliated Hospital of Chongqing Medical University, Chongqing, China

**Keywords:** extensive-stage small cell lung cancer, predictive biomarkers, prognostic factors, fibrinogen, gastrin-releasing peptide precursor

## Abstract

**Introduction:**

Extensive-stage small cell lung cancer (ES-SCLC) is an aggressive malignancy with a poor prognosis. This study aimed to identify and validate clinical and laboratory biomarkers for predicting treatment response and overall survival (OS) in ES-SCLC patients.

**Methods:**

We retrospectively analyzed 101 ES-SCLC patients receiving first-line treatment. Logistic and Cox regression analyses identified independent factors influencing treatment efficacy and OS. Subgroup analysis was performed to compare white blood cell (WBC) changes between chemotherapy-alone and chemo-immunotherapy groups. Predictive models were constructed and evaluated via cross-validation, ROC, and calibration curves. Differential expression of key proteins (neuron-specific enolase (NSE), fibrinogen (FIB), and gastrin-releasing peptide precursor (ProGRP)) was validated using GEO database data.

**Results:**

Pre-chemotherapy tumor size and post-cycle 2 FIB levels were independent predictors of treatment efficacy. Pre-chemotherapy WBC count, pre-chemotherapy D-dimer, and post-cycle 2 ProGRP were independent risk factors for OS. The predictive models demonstrated strong performance. Subgroup analysis showed no significant difference in WBC changes between treatment regimens (mean change: -2.30 ± 2.47 vs. -2.08 ± 2.45, p=0.659). GEO data confirmed the differential expression of FIB and ProGRP.

**Discussion:**

Our findings establish robust and validated models based on readily available clinical metrics (tumor size, WBC, D-dimer, FIB, ProGRP) to predict outcomes in ES-SCLC, which could aid in personalizing treatment strategies. The stability of WBC trends across therapies strengthens the prognostic value of baseline WBC.

## 1 Introduction

Small cell lung cancer (SCLC) is a highly aggressive neuroendocrine malignancy that accounts for approximately 15% of all lung cancer cases. SCLC is classified into limited-stage (LS-SCLC) and extensive-stage (ES-SCLC) according to the Veterans Administration Lung Study Group (VALSG) staging system. While LS-SCLC patients are typically treated with concurrent chemoradiotherapy, ES-SCLC patients, who comprise approximately 70% of all SCLC cases, receive systemic therapy with chemotherapy or chemotherapy combined with immunotherapy as the standard first-line treatment. Characterized by its rapid growth and early metastasis, ES-SCLC often presents at an advanced stage, making it more difficult to treat compared to non-small cell lung cancer (NSCLC) (1). Despite initial sensitivity to chemotherapy and radiotherapy, most patients diagnosed with ES-SCLC ultimately face a grim prognosis due to the disease’s tendency to progress rapidly. In the early stages, ES-SCLC often responds well to standard treatment regimens, which typically include a combination of cisplatin or carboplatin-based chemotherapy and radiation therapy. However, this initial responsiveness can be misleading. A significant proportion of these patients will encounter disease recurrence, which is characterized by the re-emergence of cancer that is not only more aggressive but also increasingly resistant to the same therapeutic approaches that initially seemed effective. This phenomenon of treatment resistance is largely attributed to the cancer cell to undergo genetic and phenotypic changes, enabling it to circumvent the mechanisms of action of chemotherapy and radiotherapy. The cumulative effect of these factors contributes to the overall poor prognosis associated with small cell lung cancer, illustrating a challenging landscape for both patients and healthcare providers (2). The median overall survival for ES-SCLC patients is only 10–12 months, with a 5-year survival rate of less than 7% (3). These stark clinical realities underscore the critical need to bridge the translational gap between biomarker discovery and therapeutic decision-making in ES-SCLC management.

Previous studies have indicated that various serum markers may play a significant role in predicting the prognosis of patients with ES-SCLC. Among these markers, neuron-specific enolase (NSE), fibrinogen (FIB), and gastrin-releasing peptide precursor (GRPP) have garnered particular attention due to their potential to provide valuable insights into disease progression and patient outcomes (4, 5). NSE is a specialized glycolytic enzyme that is predominantly expressed in neuroendocrine cells, which are a type of cell that produces hormones and neurotransmitters in the body. Due to its significant role and expression in these cells, NSE has gained prominence as a biomarker in the context of various neuroendocrine tumors, particularly SCLC (6). Elevated levels of NSE in patients with SCLC have consistently been linked to poor prognosis and treatment resistance, underscoring its importance as a biomarker in the clinical management of this aggressive form of cancer. Research indicates that when NSE levels are elevated, it often reflects a more advanced stage of the disease, characterized by increased tumor burden and more extensive metastasis (7). FIB is a critical protein in the coagulation cascade, serving as the precursor to fibrin, which plays a vital role in blood clot formation. However, beyond its essential function in hemostasis, emerging research has demonstrated that fibrinogen is also implicated in various pathological processes, including tumor growth, angiogenesis, and metastasis across a range of malignancies, including lung cancer (8). Gastrin-releasing peptide precursor, also known as pro-gastrin-releasing peptide (ProGRP), is a neuropeptide that has been found to be specifically elevated in SCLC patients and correlates with disease extent and prognosis (9, 10).

However, the prognostic value of these biomarkers in predicting treatment efficacy and survival in ES-SCLC patients remains controversial, and their clinical utility is limited by the lack of robust predictive models. Moreover, the differential expression patterns of these biomarkers in ES-SCLC tissues compared to normal lung tissues have not been thoroughly investigated. This knowledge gap presents a pivotal opportunity to develop integrative prognostic frameworks that combine molecular biomarkers with clinicopathological parameters, which could fundamentally advance risk stratification in ES-SCLC.

## 2 Methods

### 2.1 Study population

A total of 101 patients diagnosed with extensive-stage small cell lung cancer were enrolled in this study based on predefined inclusion and exclusion criteria. The inclusion criteria were as follows: (1) patients with histologically or cytologically confirmed ES-SCLC; (2) extensive-stage disease defined as disease beyond the ipsilateral hemithorax, including malignant pleural or pericardial effusion or hematogenous metastases according to VALSG criteria; (3) patients aged 18 years or older; (4) patients with an Eastern Cooperative Oncology Group (ECOG) performance status of 0-2; and (5) patients receiving first-line systemic treatment (chemotherapy alone or chemotherapy combined with immunotherapy).

The exclusion criteria were as follows: (1) patients with a history of other malignancies; (2) patients with severe comorbidities; (3) patients with incomplete clinical data; (4) patients with limited-stage disease.

The study was approved by the Institutional Review Board. This study obtained a waiver of informed consent from the hospital’s ethics committee. The ethics approval document/letter is provided as **Supplementary Material**.

### 2.2 Clinical data collection

#### 2.2.1 Efficacy assessment

Treatment efficacy was assessed after two cycles of first-line chemotherapy according to the Response Evaluation Criteria in Solid Tumors (RECIST) version 1.1. Patients were classified into two groups based on their treatment efficacy: effective (complete response or partial response) and ineffective (stable disease or progressive disease). The overall effective treatment rate was calculated as the proportion of patients with effective outcomes among the total study population.

#### 2.2.2 Clinical characteristics collection

Baseline characteristics, including age, gender, tumor size, metastasis status, and treatment regimens, were collected from the medical records of ES-SCLC patients. Tumor size was measured using computed tomography (CT) or magnetic resonance imaging (MRI) before the initiation of chemotherapy. Metastatic organ involvement was evaluated using imaging techniques such as CT, MRI, or positron emission tomography (PET) scans. Treatment regimens were recorded as either chemotherapy alone or a combination of chemotherapy and immunotherapy.

#### 2.2.3 Sample size

The sample size was calculated based on the receiver operating characteristic (ROC) curve analysis, with the area under curve (AUC) as the primary outcome measure. Assuming an expected AUC of 0.80 for the predictive model (clinically meaningful discrimination), a null hypothesis AUC of 0.50 (no discrimination), 80% statistical power (β = 0.2) and a two-sided significance level of α = 0.05, the minimum required sample size was calculated using the following formula


n=(Zα+Zβ)^2 * AUC(1−AUC)/(AUC−0.5)^2

The final sample size of 100 patients was enrolled. This sample size provided 75% power to detect an AUC ≥0.75.

#### 2.2.4 Laboratory examination collection

Prior to the initiation of first-line chemotherapy and following the completion of two treatment cycles, a panel of laboratory indicators was assessed to evaluate potential changes associated with treatment. This panel included neuron-specific enolase (NSE), fibrinogen (FIB), gastrin-releasing peptide precursor (Pro-GRP), white blood cell (WBC) count, and D-dimer levels.

Venous blood samples were collected from patients at the specified time points. Complete blood counts, including WBC, were obtained using a BC6900 hematology analyzer (Shenzhen Mindray Bio-Medical Electronics Co., Ltd., Shenzhen, China) with reagents from the same manufacturer. Fibrinogen and D-dimer levels were quantified using a STA-R-MAX coagulation analyzer (Diagnostica Stago, Asnières-sur-Seine, France) with reagents from Diagnostica Stago. NSE levels were determined using an electrochemiluminescence immunoassay on a Roche cobas 8000 e801 analyzer (Roche Diagnostics GmbH, Mannheim, Germany) with Elesys NSE reagents (Roche Diagnostics GmbH). Pro-GRP levels were determined using commercially available ELISA kits (Fujirebio, Cat. No. E-4340) and measured with an ELISA instrument (BioTek ELx800). All assays were performed according to the manufacturers’ instructions.

### 2.3 Gene expression omnibus database acquisition and analysis

To validate the differential expression levels of NSE, FIB, and gastrin-releasing peptide precursor in SCLC patients, the research team utilized the GEO database. The SCLC patient dataset (GSE149507) was obtained from the GEO database, which included gene expression data from both normal lung tissues and SCLC tissues. The raw data were processed and normalized using the robust multi-array average (RMA) method. The expression levels of NSE, FIB, and gastrin-releasing peptide precursor were compared between normal tissues and SCLC tissues using the Student’s t-test or Mann-Whitney U test, depending on the data distribution. A P-value of less than 0.05 was considered statistically significant.

### 2.4 Statistical analysis

All statistical analyses were performed using R software (version 4.0.3) and appropriate packages. A P-value of less than 0.05 was considered statistically significant, except where otherwise specified. Continuous variables were expressed as mean ± standard deviation or median (interquartile range), while categorical variables were presented as frequencies and percentages. The chi-square test or Fisher’s exact test was used to compare categorical variables between groups, while the Student’s t-test or Mann-Whitney U test was employed for continuous variables. To address potential confounding effects of treatment regimen on laboratory parameters, subgroup analyses were performed comparing WBC changes between patients receiving chemotherapy alone versus chemotherapy plus immunotherapy. The comparison was conducted using independent t-tests for absolute WBC changes and Wilcoxon signed-rank tests for paired comparisons within treatment arms.

Univariate and multivariate logistic regression analyses were performed to identify independent factors influencing treatment efficacy. Variables with a P-value of less than 0.1 in the univariate analysis were included in the multivariate analysis. A predictive model for treatment efficacy was established based on the results of the multivariate logistic regression analysis. The model’s performance was evaluated using internal cross-validation, and the area under the receiver operating characteristic (ROC) curve was calculated. A clinical calibration curve was plotted to assess the agreement between the predicted and actual values, and a clinical decision curve analysis was conducted to evaluate the model’s net clinical benefit.

Univariate and multivariate Cox regression analyses were performed to identify independent risk factors influencing overall survival (OS). Variables with a P-value of less than 0.1 in the univariate analysis were included in the multivariate analysis. A predictive model for OS was established based on the results of the multivariate Cox regression analysis. The model’s performance was assessed using time-dependent ROC curves and a calibration curve for 1-year survival.

## 3 Results

### 3.1 Logistic regression analysis and predictive model establishment for treatment efficacy in SCLC patients

#### 3.1.1 Comparative analysis of baseline characteristics in SCLC patients

A total of 101 patients diagnosed with ES-SCLC were enrolled in this study based on the inclusion and exclusion criteria. All patients had extensive-stage disease at diagnosis and received appropriate first-line systemic therapy. Among them, 59 patients received chemotherapy as the first-line treatment, while 42 patients received a combination of chemotherapy and immunotherapy. The detailed treatment regimens were provided in **Supplementary Table 1**. Efficacy assessment revealed that 52 patients had ineffective treatment outcomes, and 49 patients had effective outcomes. The overall effective treatment rate was 48.5% (49/101)(**Table 1**).

**Table 1:** Univariate analysis results of treatment efficacy in SCLC patients.

Characteristic	[ALL]	Ineffective Group	Effective Group	p.overall	Adjusted_P
	*N=101*	*N=49*	*N=52*		
Gender:				0.319	0.959
Female	15 (14.9%)	5 (10.2%)	10 (19.2%)		
Male	86 (85.1%)	44 (89.8%)	42 (80.8%)		
Age	62.6 ± 9.77	61.8 ± 10.1	63.4 ± 9.46	0.400	1.000
First-lineRegimen:				1.000	1.000
Chemotherapy	59 (58.4%)	29 (59.2%)	30 (57.7%)		
Chemotherapy+Immunotherapy	42 (41.6%)	20 (40.8%)	22 (42.3%)		
Pre-chemotherapyWBC	6.63 [2.46;14.9]	6.81 [2.46;12.3]	6.49 [3.41;14.9]	0.540	1.000
Pre-chemotherapyPLT	231 [73.0;519]	227 [73.0;404]	235 [101;519]	0.967	1.000
Pre-chemotherapyLym	1.24 [0.03;3.93]	1.41 [0.03;3.93]	1.19 [0.42;2.81]	0.518	1.000
Pre-chemotherapyFIB	4.34 [1.03;10.9]	4.52 [1.03;10.9]	4.14 [2.48;8.22]	0.195	1.000
Pre-chemotherapyD-dimer	192 [14.0;6190]	192 [14.0;6190]	196 [31.0;3178]	0.253	1.000
Pre-chemotherapyNSE	45.3 [7.99;525]	33.5 [10.1;370]	53.2 [7.99;525]	0.016	1.000
Pre-chemotherapyProGRP	858 [33.5;25000]	1240 [33.5;15872]	825 [44.0;25000]	0.638	1.000
Pre-chemotherapyTumorSize	12.1 [1.50;37.0]	10.0 [1.50;37.0]	14.6 [4.50;34.9]	0.001	0.007
Pre-chemotherapyMetastaticOrgans:				0.907	1.000
1	17 (16.8%)	9 (18.4%)	8 (15.4%)		
2	65 (64.4%)	31 (63.3%)	34 (65.4%)		
3	12 (11.9%)	5 (10.2%)	7 (13.5%)		
4	7 (6.93%)	4 (8.16%)	3 (5.77%)		

WBC, whitebloodcell; PLT, platelet; Lym, lymphocyte; FIB, fibrinogen; NSE, neuron-specificenolase; ProGRP, pro-gastrin-releasingpeptide.

To evaluate whether treatment regimen influenced WBC dynamics, we performed a comprehensive subgroup analysis. The results showed that the mean WBC change was -2.30 ± 2.47 × 10_9_/L in the chemotherapy alone group and -2.08 ± 2.45 × 10_9_/L in the chemotherapy plus immunotherapy group (p=0.659, independent t-test), indicating no significant difference in WBC changes between treatment regimens (**Supplementary Figure 1**, **Supplementary Table 2**).

Baseline characteristics such as age, gender, tumor size, metastasis status, and treatment regimens were collected from SCLC patients. Additionally, changes in various indicators before chemotherapy were recorded. Patients were grouped according to their treatment efficacy after two cycles, and the indicators were compared between groups. The results showed statistically significant differences in pre-chemotherapy NSE levels and tumor size (P<0.05) (**Table 1**).

### 3.2 Trends in blood cell counts during treatment in SCLC patients

Laboratory examination indicators before chemotherapy and after two cycles were compared between the two groups. The changes in these indicators from pre-treatment to post-treatment were also calculated. The results demonstrated that pre-chemotherapy NSE, post-cycle 2 FIB, post-cycle 2 gastrin-releasing peptide precursor, and NSE change values exhibited statistically significant differences between the two groups (P<0.05) (**Table 2**).

**Table 2:** Changes in laboratory tests.

Characteristic	[ALL]	Ineffective Group	Effective Group	p.overall	Adjusted_P
	*N=101*	*N=49*	*N=52*		
Pre-chemotherapyWBC	6.63 [2.46;14.9]	6.81 [2.46;12.3]	6.49 [3.41;14.9]	0.540	1.000
Pre-chemotherapyPLT	231 [73.0;519]	227 [73.0;404]	235 [101;519]	0.967	1.000
Pre-chemotherapyLym	1.24 [0.03;3.93]	1.41 [0.03;3.93]	1.19 [0.42;2.81]	0.518	1.000
Pre-chemotherapyFIB	4.34 [1.03;10.9]	4.52 [1.03;10.9]	4.14 [2.48;8.22]	0.195	1.000
Pre-chemotherapyD-dimer	192 [14.0;6190]	192 [14.0;6190]	196 [31.0;3178]	0.253	1.000
Pre-chemotherapyNSE	45.3 [7.99;525]	33.5 [10.1;370]	53.2 [7.99;525]	0.016	1.000
Pre-chemotherapyProGRP	858 [33.5;25000]	1240 [33.5;15872]	825 [44.0;25000]	0.638	1.000
Post-2-cycleWBC	4.75 [2.19;10.7]	4.76 [2.19;9.07]	4.63 [2.48;10.7]	0.883	1.000
Post-2-cyclePLT	212 [86.0;579]	215 [86.0;503]	207 [91.0;579]	0.629	1.000
Post-2-cycleLym	1.26 [0.14;3.83]	1.30 [0.14;3.83]	1.25 [0.38;2.80]	0.997	1.000
Post-2-cycleFIB	3.32 [1.09;5.82]	3.70 [1.09;5.82]	3.10 [2.02;4.06]	<0.001	0.001
Post-2-cycleD-dimer	183 [21.0;3549]	178 [21.0;2250]	194 [37.0;3549]	0.447	1.000
Post-2-cycleNSE	20.0 [1.00;81.9]	22.6 [1.00;72.6]	19.4 [5.49;81.9]	0.401	1.000
Post-2-cycleProGRP	113 [23.3;14155]	403 [31.2;12950]	75.8 [23.3;14155]	0.026	1.000
WBCchange	2.31 ± 2.46	2.36 ± 2.23	2.27 ± 2.67	0.858	1.000
PLTchange	32.3 ± 95.1	24.1 ± 97.8	40.1 ± 92.9	0.401	1.000
Lymchange	0.04 ± 0.42	0.07 ± 0.46	0.01 ± 0.39	0.485	1.000
FIBchange	0.78 [-1.00;6.73]	0.66 [-1.00;6.73]	0.80 [-0.16;4.52]	0.065	1.000
D-dimerchange	9.00 [-2084.00;6049]	20.0 [-2084.00;6049]	7.50 [-936.00;3134]	0.833	1.000
NSEchange	22.7 [-32.31;497]	13.2 [-32.31;338]	34.8 [-16.44;497]	0.007	1.000
ProGRPchange	584 [-5398.00;24946]	196 [-5398.00;10737]	678 [-3000.00;24946]	0.123	1.000

WBC, white blood cell; PLT, platelet; Lym, lymphocyte; FIB, fibrinogen; NSE, neuron-specificenolase; ProGRP, pro-gastrin-releasing peptide.

### 3.3 Multivariate logistic regression analysis of treatment efficacy in SCLC patients

**Figure 1:**
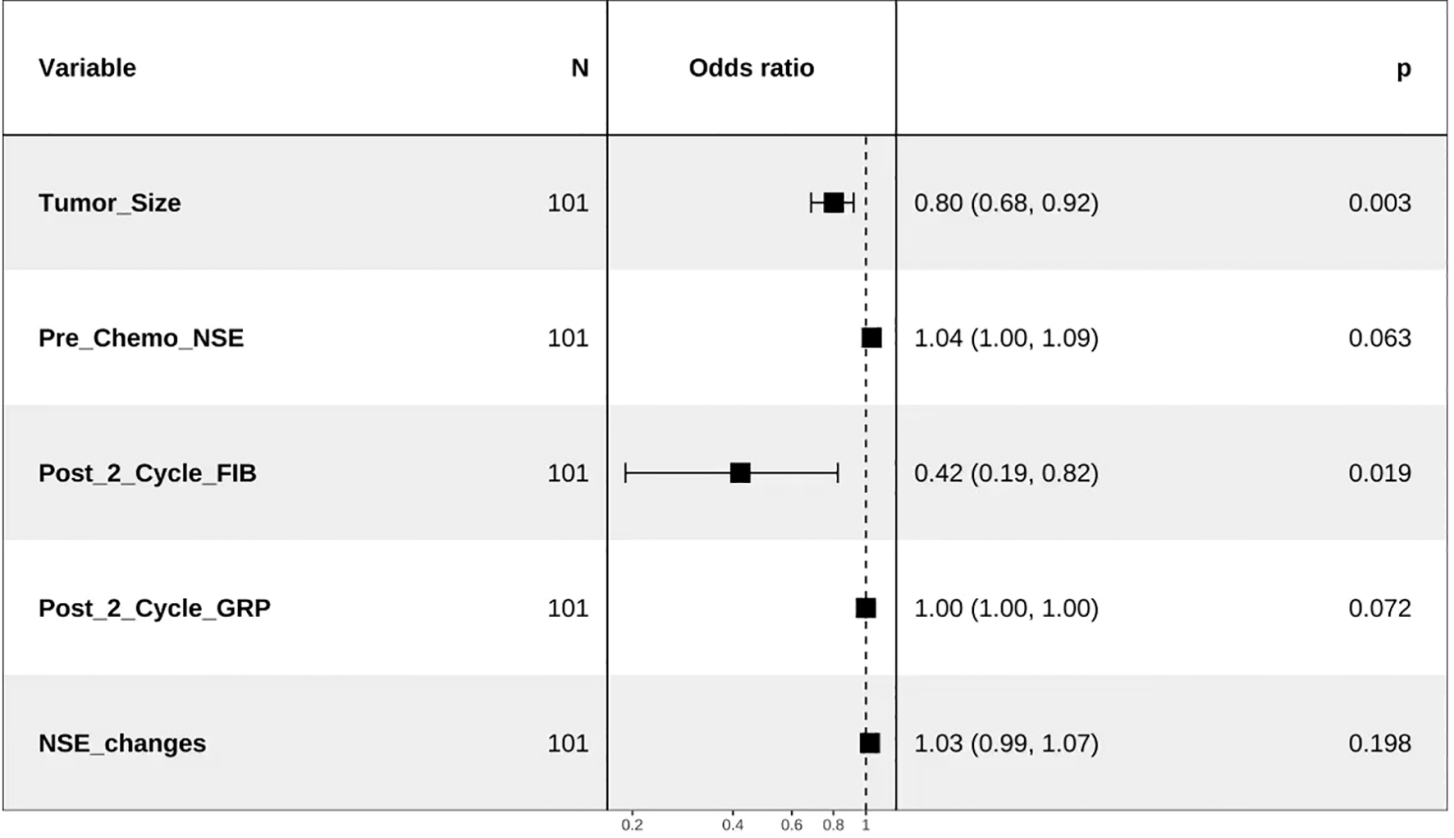
Forest plot of the multivariate regression analysis.

Tumor size before chemotherapy, pre-chemotherapy NSE, post-cycle 2 FIB, post-cycle 2 gastrin-releasing peptide precursor, and NSE change values were included in the multivariate logistic regression analysis. The results, as shown below, indicated that post-cycle 2 FIB and pre-chemotherapy tumor size were independent factors influencing the treatment efficacy in SCLC patients (P<0.05) (**Table 3**, **Figure 1**).

**Table 3:** Multivariate analysis results of treatment efficacy in SCLC patients.

Variables	Coef	S.E.	WaldZ	Pr(>|Z|)	OR
Intercept	3.1705	1.3442	2.36	0.018	23.81
Pre-chemotherapyTumorSize	0.1329	0.0451	2.95	0.003	0.80
Pre-chemotherapyNSE	0.0047	0.0223	0.21	0.063	1.04
Post-2-cycleFIB	-1.5093	0.4669	-3.23	0.019	0.42
Post-2-cycleProGRP	-0.0001	0.0001	-1.02	0.072	1.00
NSEchange	-0.0012	0.0231	-0.05	0.198	1.03

NSE, neuron-specificenolase; FIB, fibrinogen; ProGRP, pro-gastrin-releasing peptide; Coef, coefficient; S.E., standard error; OR, odds ratio.

### 3.4 Establishment and validation of a predictive model for treatment efficacy after two cycles of chemotherapy in SCLC patients

**Figure 2:**
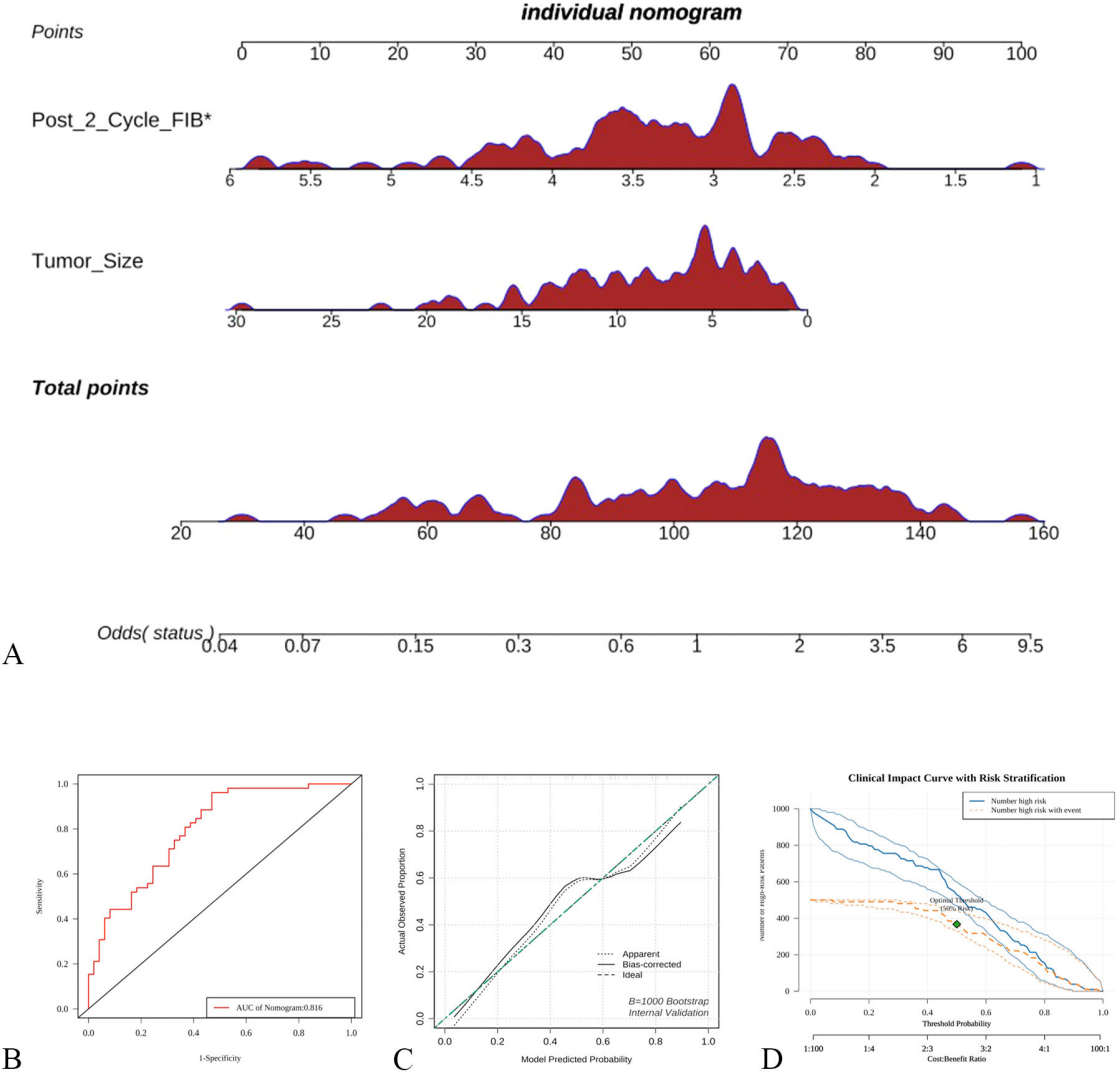
Establishment and validation of a predictive model based on logistic regression **(A)** nomogram based on logistic regression, **(B)** ROC curve; **(C)** Calibration curve, **(D)** Clinical influence curve.

Based on the results of the multivariate logistic regression analysis, a predictive model was established using pre-chemotherapy tumor size and post-cycle 2 FIB as risk factors. Nomogram was plotted to illustrate the model (**Figure 2A**). Internal cross-validation was performed, and the area under the ROC curve was calculated to be 0.816 (**Figure 2B**). The clinical calibration (**Figure 2C**) curve demonstrated good agreement between the predicted and actual values. The clinical influence curve analysis (**Figure 2D**) showed that the model had a favorable net clinical benefit.

### 3.5 Cox regression analysis and predictive model establishment for treatment efficacy in SCLC patients

#### 3.5.1 Univariate Cox regression analysis

**Figure 3:**
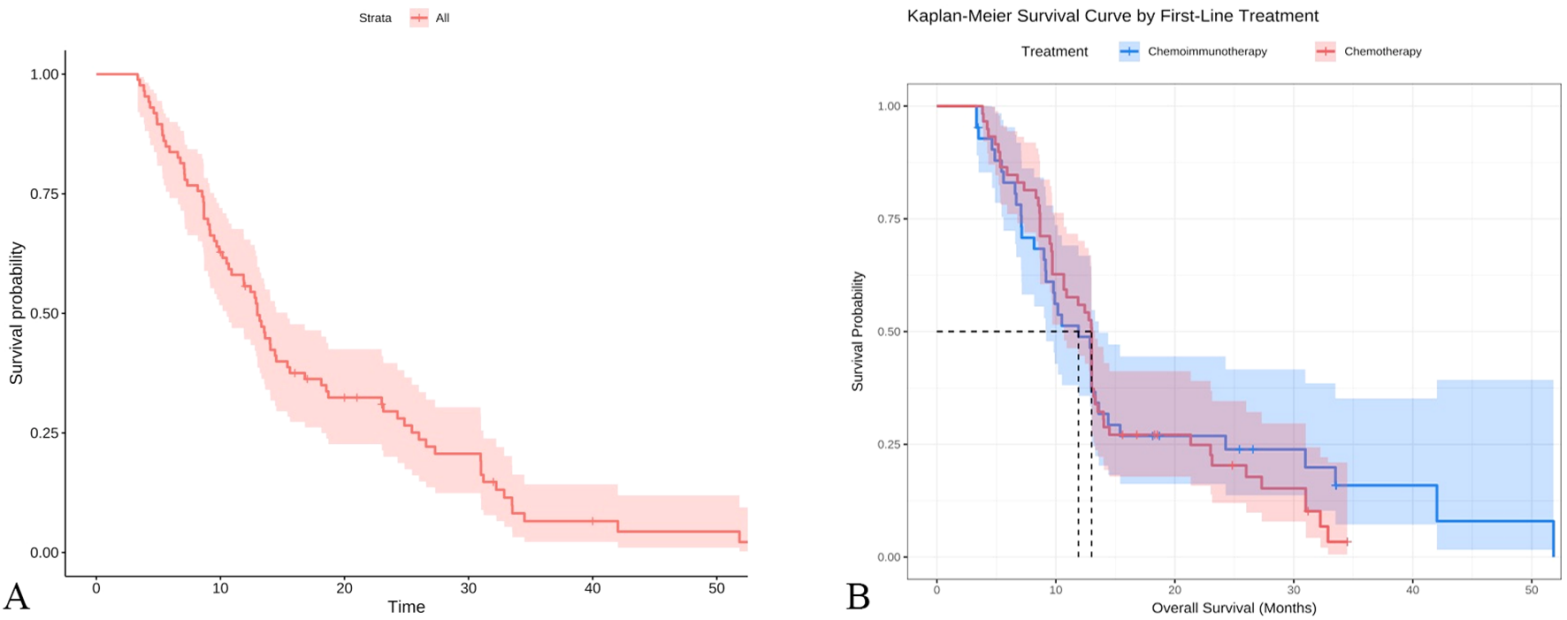
Survival curve analysis of SCLC patients **(A)** Kaplan-Meier analysis of all patients; **(B)** Subgroup Kaplan-Meier analysis by first-line treatment regimen.

A total of 101 SCLC patients were included in this study. Long-term follow-up results showed that 87 subjects died, while 14 were still alive at the last follow-up. The median overall survival (OS) of SCLC patients was calculated to be 13 months (95% CI: 10.5-15.4 months)(**Figure 3A**).Subgroup analysis based on the first-line treatment regimen showed no significant difference in OS between chemotherapy and chemotherapy combined with immunotherapy(**Figure 3B**). Univariate Cox regression analysis was performed based on OS status, time variables, and various data for each patient. The results demonstrated significant differences in pre-chemotherapy tumor size, pre-chemotherapy WBC, pre-chemotherapy NSE, pre-chemotherapy gastrin-releasing peptide precursor, pre-treatment FIB, post-cycle 2 D-dimer, post-cycle 2 gastrin-releasing peptide precursor, NSE change value, and WBC change value (P<0.05) (**Table 4**).

**Table 4:** COX analysis results of treatment efficacy in SCLC patients.

Variables	Coef	S.E.	Wald Z	Pr(>|Z|)	HR
Gender=Male	0.1488	0.3286	0.45	0.650	1.16
First-line=Chemo+Immuno	-0.1722	0.2363	-0.73	0.466	0.84
Pre-chemo Tumor Size	0.0644	0.0175	3.69	<0.001	1.06
Pre-chemo WBC	0.1399	0.0476	2.94	0.003	1.15
Pre-chemo PLT	0.0021	0.0013	1.59	0.112	1.00
Pre-chemo Lym	-0.0585	0.2073	-0.28	0.777	0.94
Pre-chemo NSE	0.0063	0.0014	4.4	<0.0001	1.00
Pre-chemo ProGRP	0.0001	0	2.4	0.016	1.00
Pre-chemo FIB	0.2468	0.0861	2.87	0.004	1.27
Pre-chemo D-dimer	0.0002	0.0001	2.4	0.016	1.00
Post-2-cycle Lym	0.1776	0.2153	0.83	0.409	1.19
Post-2-cycle FIB	0.2527	0.1382	1.83	0.067	1.28
Post-2-cycle D-dimer	0.0006	0.0002	2.4	0.016	1.00
Post-2-cycle NSE	0.0118	0.0077	1.53	0.125	1.01
Post-2-cycle ProGRP	0.0001	0	3.47	<0.001	1.00
FIB change	0.1893	0.098	1.93	0.053	1.20
D-dimer change	0.0002	0.0001	1.55	0.120	1.00
NSE change	0.0064	0.0015	4.33	<0.0001	1.00
ProGRP change	0	0	0.97	0.334	1.00
WBC change	0.101	0.0457	2.23	0.025	1.10
PLT change	0.0015	0.0014	1	0.315	1.00
Lym change	-0.3615	0.2732	-1.32	0.185	0.69

WBC, white blood cell; PLT, platelet; Lym, lymphocyte; FIB, fibrinogen; NSE, neuron-specific enolase; ProGRP, pro-gastrin-releasing peptide; Coef, coefficient; S.E., standard error; HR, hazard ratio.

#### 3.5.2 Multivariate Cox regression analysis

Multivariate Cox regression analysis was conducted based on pre-chemotherapy tumor size, pre-chemotherapy WBC, pre-chemotherapy NSE, pre-chemotherapy gastrin-releasing peptide precursor, pre-treatment FIB, post-cycle 2 D-dimer, post-cycle 2 gastrin-releasing peptide precursor, NSE change value, and WBC change value. The results indicated that pre-chemotherapy WBC, pre-chemotherapy D-dimer, and post-cycle 2 gastrin-releasing peptide precursor were independent risk factors influencing OS (P<0.05) (**Table 5**).

**Table 5:** Multivariate COX analysis results of treatment efficacy in SCLC patients.

Variables	Coef	S.E.	Wald Z	Pr(>|Z|)	HR
Pre-chemo Tumor Size	0.0321	0.0205	1.56	0.118	1.032
Pre-chemo WBC	0.1415	0.0769	1.84	0.035	1.15
Pre-chemo NSE	0.0009	0.0088	0.11	0.914	1.00
Pre-chemo ProGRP	0	0	-0.34	0.730	0.99
Pre-chemo FIB	0.0616	0.1051	0.59	0.557	1.06
Pre-chemo D-dimer	0.0003	0.0001	1.84	0.046	1.00
Post-2-cycle D-dimer	0.0003	0.0003	1.14	0.253	1.00
Post-2-cycle ProGRP	0.0001	0.0001	2.06	0.039	1.00
NSE change	0.0036	0.0095	0.37	0.708	1.00
WBC change	-0.0214	0.0697	-0.31	0.759	0.97

WBC, white blood cell; FIB, fibrinogen; NSE, neuron-specific enolase; ProGRP, pro-gastrin-releasing peptide; Coef, coefficient; S.E., standard error; HR, hazard ratio.

##### 3.5.2.1 Establishment of a predictive model based on multivariate Cox regression analysis

**Figure 4:**
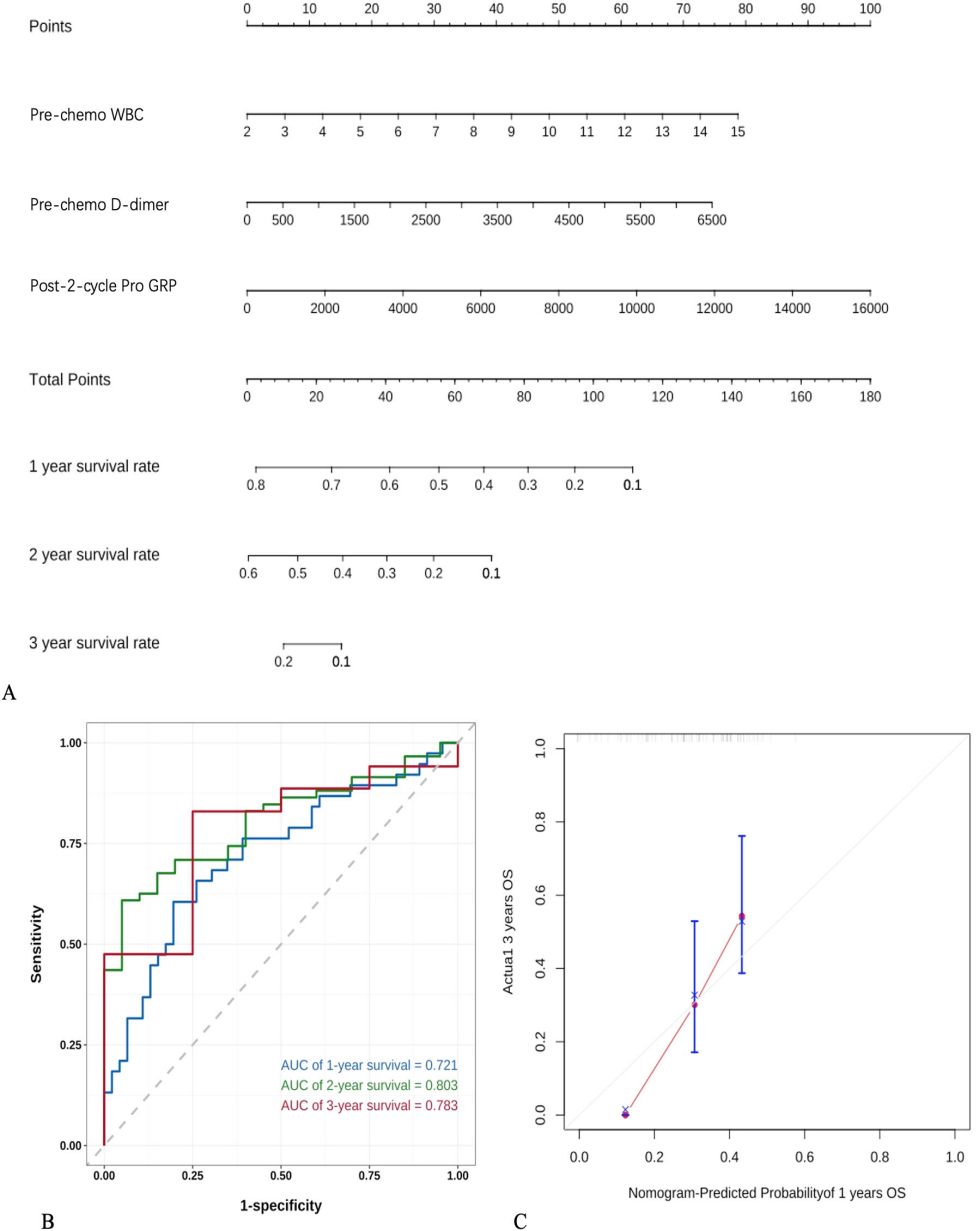
Establishment and validation of a predictive model based on logistic regression **(A)** nomogram based on COX regression, **(B)** Time-ROC curve, **(C)** Calibration curve.

A predictive model was established based on pre-chemotherapy WBC, pre-chemotherapy D-dimer, and post-cycle 2 gastrin-releasing peptide precursor, as shown in the figure below(**Figure 4A**). Internal validation was performed by plotting time-dependent ROC curves, which demonstrated that the model had good predictive value for 1-year, 2.5-year, and 3-year survival rates(**Figure 4B**). A calibration curve for 1-year survival was also plotted, showing good agreement between the predicted and actual values (**Figure 4C**).

### 3.6 Differential expression analysis of NSE, FIB, and gastrin-releasing peptide precursor in SCLC patients

**Figure 5:**
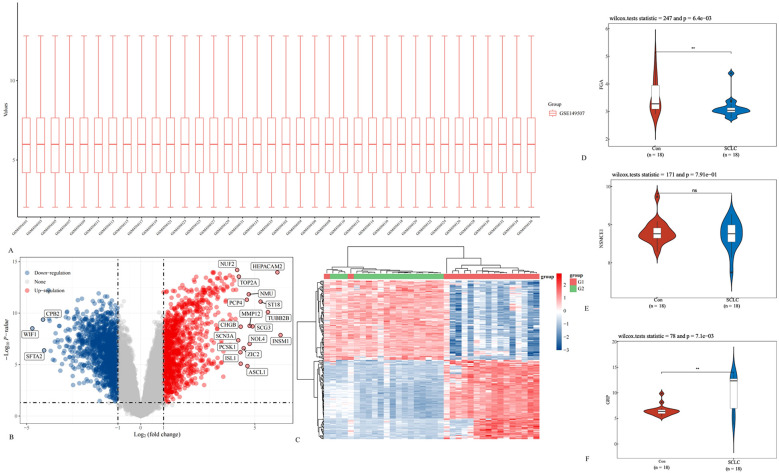
Differential expression analysis of NSE, FIB, and gastrin-releasing peptide precursor in SCLC patients. **(A)** Box plot displaying the batch-normalized gene expression distribution of the GSE149507 dataset; **(B)** volcano plot of DEGs; **(C)** heat map of DEGs; **(D)** FGA, **(E)** NSMCE1, and **(F)** GRP expression comparisons between the SCLC and control groups.

**Figure 6:**
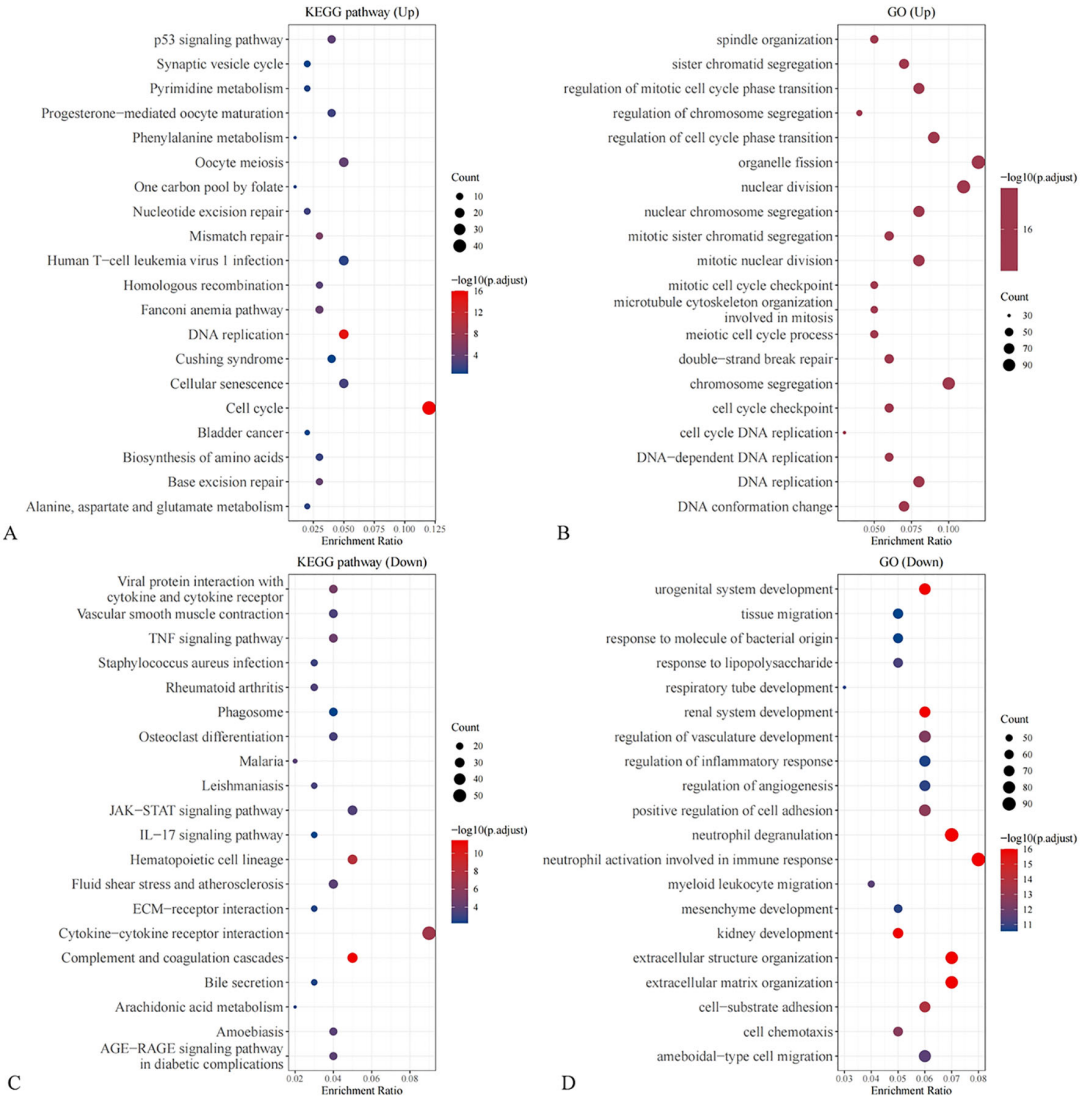
Enrichment analysis of DEGs **(A)** KEGG enrichment based on up-regulated DEGs; **(B)** GO enrichment based on up-regulated DEGs; **(C)** KEGG enrichment based on down-regulated DEGs; **(D)** GO enrichment based on down-regulated DEGs.

Previous clinical studies have preliminarily identified neuron-specific enolase (NSE), fibrinogen (FIB), and gastrin-releasing peptide precursor as key protein molecules that influence the treatment efficacy and prognosis of small cell lung cancer (SCLC) patients. Based on these findings, the research team conducted an external validation of the differential expression levels of NSE, FIB, and gastrin-releasing peptide precursor using the Gene Expression Omnibus (GEO) database. The SCLC patient dataset (GSE149507) was obtained from the GEO database, and the expression levels of NSE, FIB, and gastrin-releasing peptide precursor were compared between normal tissues and SCLC tissues. The results showed that both FIB and gastrin-releasing peptide precursor exhibited significant differential expression in SCLC tissues compared to normal tissues(**Figure 5**). GO and KEGGenrichment analyses were performed based on the DEGs (**Figure 6**).

**Figure 7:**
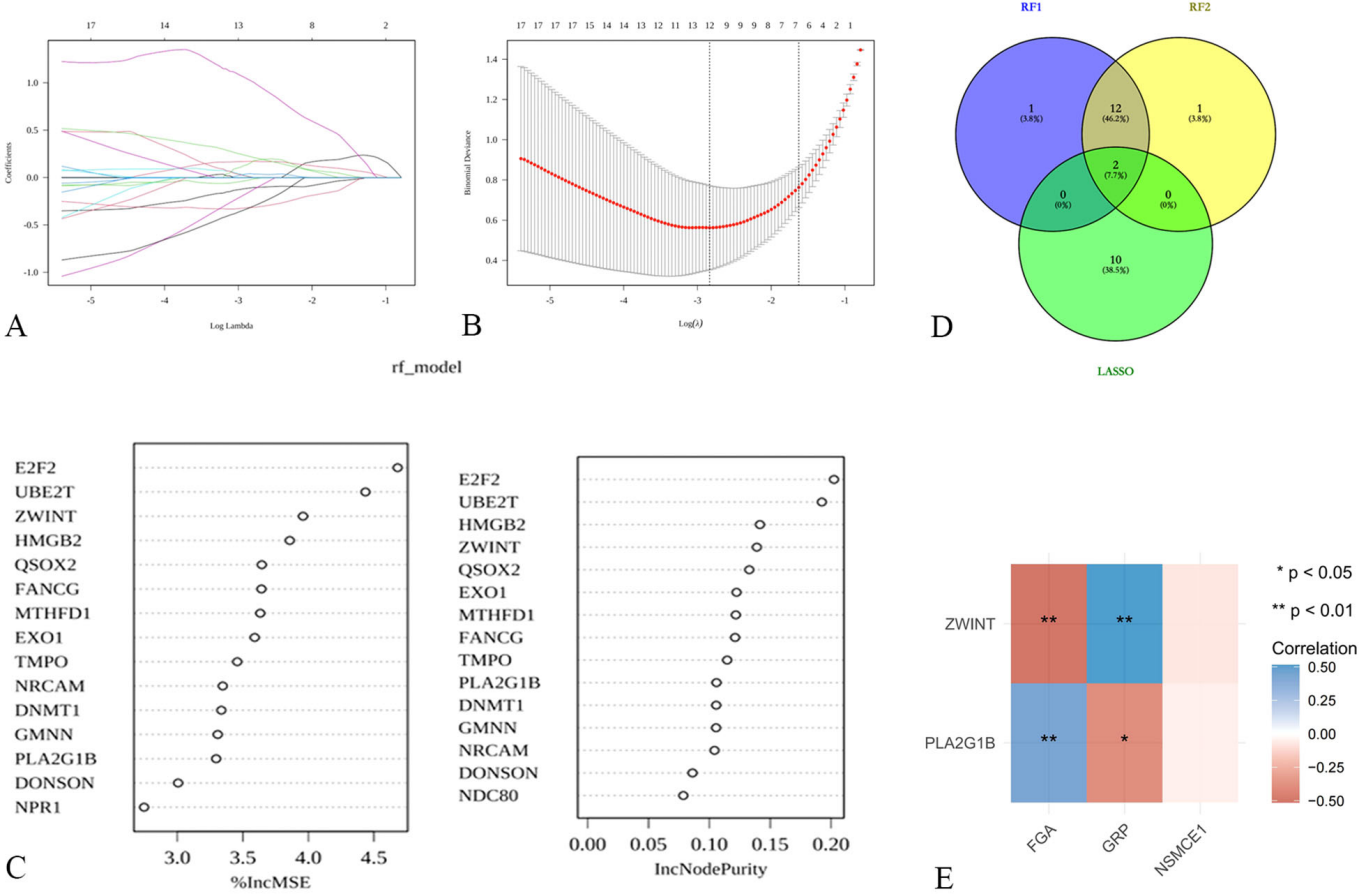
Exploration of potential mechanisms of hub targets in SCLC **(A, B)** LASSO regression for screening core targets; **(C)** Identification of core targets using random forest; **(D)** Venn diagram for determining shared core targets; **(E)** Correlation analysis between shared core targets and FGA, NSMCE1, and GRP.

To further investigate the mechanisms by which NSE, FIB, and ProGRP mediate SCLC, differentially expressed genes (DEGs) in SCLC were identified. LASSO regression analysis (**Figures 7A, B**) and random forest analysis (**Figure 7C**) were performed. The intersection of these analyses yielded two core target genes. Correlation analysis revealed that these two core target genes (ZWINT and PLA2G1B) were significantly correlated with FIB and ProGRP (**Figure 7D**).

## 4 Discussion

Extensive-stage Small cell lung cancer (ES-SCLC) is a highly aggressive malignancy characterized by rapid progression, early metastasis, and poor prognosis (1). Despite initial sensitivity to chemotherapy, most ES-SCLC patients experience relapse and develop drug resistance, leading to treatment failure and limited survival. Therefore, identifying reliable predictive biomarkers and establishing prognostic models are crucial for optimizing treatment strategies and improving patient outcomes in ES-SCLC.

In this study, we conducted a comprehensive analysis of clinical data and laboratory indicators to investigate their potential as predictive and prognostic factors in patients diagnosed with ES-SCLC. All patients enrolled in our study had extensive-stage disease and received standard first-line treatment according to current guidelines. Our research aimed to understand how various clinical and laboratory parameters could influence treatment outcomes and overall survival in this challenging cohort of patients. Through our analysis, we identified several key findings that illuminated the relationship between specific biomarkers and patient prognosis. Notably, we found that the size of the tumor prior to the initiation of chemotherapy was a significant predictive factor for treatment efficacy. This suggests that larger tumors may be associated with poorer responses to chemotherapy, thereby impacting the overall effectiveness of treatment strategies employed for ES-SCLC. Furthermore, our investigation into laboratory indicators revealed that levels of FIB measured post-cycle 2 of chemotherapy functioned as an independent factor affecting treatment efficacy. Elevated fibrinogen levels may indicate a hypercoagulable state or systemic inflammation, both of which could potentially influence the effectiveness of chemotherapeutic agents. In addition, we also examined parameters linked to OS in SCLC patients. Our results indicated that the pre-chemotherapy WBC count was an independent risk factor for overall survival. Higher WBC counts may reflect an underlying inflammatory response or tumor burden, which could negatively impact patient outcomes. Moreover, our analysis highlighted pre-chemotherapy D-dimer levels and post-cycle 2 levels of gastrin-releasing peptide precursor as additional independent risk factors for OS and found that pre-chemotherapy D-dimer, and post-cycle 2 gastrin-releasing peptide precursor were independent risk factors for OS. Emerging evidence from lung adenocarcinoma studies (11) demonstrates that thrombin, the catalytic product of fibrinogen, can cleave epidermal growth factor receptor (EGFR) to activate AKT/mTOR signaling pathways, providing a direct molecular mechanism linking coagulation activation to chemotherapy resistance. This mechanism may explain our observation of FIB’s predictive value, suggesting thrombin-mediated pathways as potential therapeutic targets in ES-SCLC.

Tumor size has been previously reported as a prognostic factor in ES-SCLC. A study by demonstrated that tumor size >5 cm was associated with worse OS in SCLC patients (12). Similarly, our results indicated that larger pre-chemotherapy tumor size was an independent predictor of poor treatment efficacy. This finding suggests that tumor burden plays a critical role in determining treatment response and highlights the importance of early diagnosis and timely intervention in ES-SCLC.

Fibrinogen, an acute-phase protein, has been implicated in cancer progression and metastasis (13). Elevated FIB levels have been associated with poor prognosis in various malignancies, including lung cancer (14). In our study, higher post-cycle 2 FIB levels were identified as an independent factor for ineffective treatment outcomes. This observation is consistent with previous reports and underscores the potential of FIB as a predictive biomarker in ES-SCLC. The groundbreaking discovery that thrombin (activated from fibrinogen) mediates chemotherapy resistance through EGFR cleavage (11) provides mechanistic support for our findings. This coagulation-tumor interaction paradigm suggests that real-time monitoring of coagulation parameters could guide anti-resistance therapies. The underlying mechanisms linking FIB to treatment resistance may involve its role in promoting tumor cell survival, angiogenesis, and epithelial-mesenchymal transition (15, 16).

White blood cell count, a marker of systemic inflammation, has been associated with poor prognosis in several cancer types (17). In SCLC, elevated WBC count has been reported as an adverse prognostic factor (18). Our findings corroborate these observations, as higher pre-chemotherapy WBC count was identified as an independent risk factor for reduced OS. The prognostic value of WBC count may be attributed to its reflection of the tumor-promoting inflammatory microenvironment and its association with increased tumor burden (19).

D-dimer, a fibrin degradation product, has been recognized as a prognostic marker in various malignancies, including lung cancer (20). Elevated D-dimer levels have been linked to increased risk of thromboembolism and worse survival outcomes (21, 22). Recent mechanistic insights (11) reveal that coagulation system activation is not merely a bystander phenomenon but actively contributes to tumor progression through EGFR signaling modulation. This biological framework strengthens the clinical significance of our D-dimer findings, positioning coagulation markers as both prognostic indicators and potential therapeutic nodes. In our study, higher pre-chemotherapy D-dimer levels were found to be an independent predictor of shorter OS in ES-SCLC patients. This finding highlights the importance of monitoring coagulation parameters and considering thromboprophylaxis in high-risk ES-SCLC patients.

Gastrin-releasing peptide precursor, a neuropeptide involved in cell proliferation and survival, has been implicated in the pathogenesis of SCLC (23). Elevated levels of gastrin-releasing peptide precursor have been associated with tumor progression and poor prognosis in SCLC (24). Our results showed that higher post-cycle 2 gastrin-releasing peptide precursor levels were an independent risk factor for reduced OS. This finding suggests that monitoring gastrin-releasing peptide precursor levels during treatment may provide valuable prognostic information and guide therapeutic decision-making in ES-SCLC.

To validate the differential expression of neuron-specific enolase (NSE), FIB, and gastrin-releasing peptide precursor in ES-SCLC, we analyzed data from the Gene Expression Omnibus (GEO) database. The results confirmed the significant upregulation of FIB and gastrin-releasing peptide precursor in ES-SCLC tissues compared to normal tissues. These findings support the potential utility of these molecules as diagnostic and prognostic biomarkers in ES-SCLC.

Based on the identified independent predictors, we established predictive models for treatment efficacy and OS in ES-SCLC patients. The model for treatment efficacy, incorporating pre-chemotherapy tumor size and post-cycle 2 FIB levels, demonstrated good predictive performance, with an area under the ROC curve of 0.8163. The model for OS, based on pre-chemotherapy WBC count, pre-chemotherapy D-dimer, and post-cycle 2 gastrin-releasing peptide precursor, exhibited satisfactory predictive value for 1-year, 2.5-year, and 3-year survival rates. These models exemplify the emerging “Clinlabomics” approach (25), where systematic integration of laboratory parameters with machine learning generates clinically actionable predictive tools. The success of our multivariable models underscores the importance of analyzing laboratory indicators as interconnected biological networks rather than isolated values. These models may serve as valuable tools for risk stratification and personalized treatment planning in ES-SCLC patients.

However, our study has several limitations. First, the sample size was relatively small, and the findings need to be validated in larger prospective cohorts. Second, the mechanisms underlying the prognostic significance of the identified biomarkers require further investigation. Third, the integration of molecular profiling data, such as genomic and transcriptomic information, may provide additional insights into the biological processes driving treatment response and prognosis in ES-SCLC. A further limitation lies in the absence of external validation. While the predictive model underwent internal cross-validation, it was not validated using an independent external dataset.

## 5 Conclusion

In conclusion, our study identified pre-chemotherapy tumor size and post-cycle 2 FIB levels as independent predictors of treatment efficacy, and pre-chemotherapy WBC count, pre-chemotherapy D-dimer, and post-cycle 2 gastrin-releasing peptide precursor as independent risk factors for OS in ES-SCLC patients receiving first-line systemic therapy. The established predictive models showed promising performance and may assist in risk stratification and treatment decision-making. Furthermore, the differential expression of FIB and gastrin-releasing peptide precursor in ES-SCLC tissues was validated using GEO data. These findings contribute to a better understanding of the factors influencing treatment outcomes and prognosis in ES-SCLC and highlight potential biomarkers for future research and clinical application. However, further validation and mechanistic studies are warranted to fully elucidate the role of these biomarkers in ES-SCLC management.

## Data Availability

The original contributions presented in the study are included in the article/**Supplementary Material**. Further inquiries can be directed to the corresponding author.
