# Supplementary material for: A predictive model and mechanistic study of treatment effectiveness in patients newly diagnosed with small cell lung cancer
Source: Front Oncol. 2025 Sep 11;15:1631490. doi: 10.3389/fonc.2025.1631490 (PMC12460094; doi:10.3389/fonc.2025.1631490)
Supplement: Supplementary file 1 [file Table1.docx]

**Supplementary Table 1. Clinical Treatment Regimens for SCLC Patients**

| **Treatment Regimen Category** | **Specific Regimen** | **N** | **Percentage (%)** |
| --- | --- | --- | --- |
| ****Chemotherapy**** |  | ****59**** | ****58.40%**** |
|  | Etoposide + Nedaplatin | 39 | 38.2% |
|  | Etoposide + Lobaplatin | 8 | 7.8% |
|  | Etoposide + Cisplatin | 9 | 8.9% |
|  | Albumin-bound paclitaxel + Carboplatin | 1 | 1.0% |
|  | Irinotecan + Nedaplatin | 1 | 1.0% |
|  | Etoposide | 1 | 1.0% |
|  | Etoposide + Carboplatin | 1 | 1.0% |
| ****Chemotherapy plus Immunotherapy**** |  | ****42**** | ****41.60%**** |
|  | Sintilimab + Etoposide + Cisplatin | 5 | 5.0% |
|  | Atezolizumab + Carboplatin + Etoposide | 4 | 4.0% |
|  | Durvalumab + Etoposide + Lobaplatin | 4 | 4.0% |
|  | Durvalumab + Etoposide + Carboplatin | 3 | 3.0% |
|  | Serplulimab + Etoposide + Cisplatin | 3 | 3.0% |
|  | Tislelizumab + Etoposide | 3 | 3.0% |
|  | Atezolizumab + Etoposide + Carboplatin | 2 | 2.0% |
|  | Atezolizumab + Etoposide + Nedaplatin | 2 | 2.0% |
|  | Sintilimab + Etoposide + Lobaplatin | 2 | 2.0% |
|  | Atezolizumab + Cisplatin | 1 | 1.0% |
|  | Atezolizumab + Cisplatin + Etoposide | 1 | 1.0% |
|  | Atezolizumab injection + Etoposide + Carboplatin | 1 | 1.0% |
|  | Durvalumab + Etoposide + Lobaplatin | 1 | 1.0% |
|  | Envafolimab + Etoposide | 1 | 1.0% |
|  | Serplulimab + Etoposide + Lobaplatin | 1 | 1.0% |
|  | Serplulimab + Etoposide injection + Cisplatin | 1 | 1.0% |
|  | Toripalimab + Irinotecan + Nedaplatin | 1 | 1.0% |
|  | Toripalimab + Etoposide + Cisplatin | 1 | 1.0% |
|  | Tislelizumab + Etoposide + Cisplatin | 1 | 1.0% |
|  | Sintilimab + Etoposide + Nedaplatin | 1 | 1.0% |
|  | Sintilimab + Paclitaxel + Lobaplatin | 1 | 1.0% |
|  | Etoposide + Lobaplatin + Durvalumab | 1 | 1.0% |
|  | Sintilimab + Etoposide | 1 | 1.0% |
| ****Total**** |  | ****101**** | ****100.00%**** |
