# Supplementary material for: A predictive model and mechanistic study of treatment effectiveness in patients newly diagnosed with small cell lung cancer
Source: Front Oncol. 2025 Sep 11;15:1631490. doi: 10.3389/fonc.2025.1631490 (PMC12460094; doi:10.3389/fonc.2025.1631490)
Supplement: Supplementary file 2 [file Table2.docx]

**Supplemental Table 2. WBC Dynamics by Treatment Regimen in Extensive-Stage Small-Cell Lung Cancer**

| **Treatment regimen** | **n** | **Pre-chemotherapy WBC**  **(×10⁹ L⁻¹)** | **Post-cycle 2 WBC**  **(×10⁹ L⁻¹)** | **Absolute WBC change**  **(×10⁹ L⁻¹)** | **Median (IQR) WBC change**  **(×10⁹ L⁻¹)** |
| --- | --- | --- | --- | --- | --- |
| Chemo-immunotherapy | 42 | 6.92 ± 2.60 | 4.84 ± 1.37 | –2.08 ± 2.45 | –1.66 (–2.99 to –0.48) |
| Chemotherapy | 59 | 7.54 ± 2.27 | 5.23 ± 1.95 | –2.30 ± 2.47 | –2.00 (–3.92 to –0.46) |

p-value for WBC change comparison (t-test) = 0.659
