# Supplementary material for: A predictive model and mechanistic study of treatment effectiveness in patients newly diagnosed with small cell lung cancer
Source: Front Oncol. 2025 Sep 11;15:1631490. doi: 10.3389/fonc.2025.1631490 (PMC12460094; doi:10.3389/fonc.2025.1631490)
Supplement: Supplementary file 3 [file DataSheet1.docx]

**
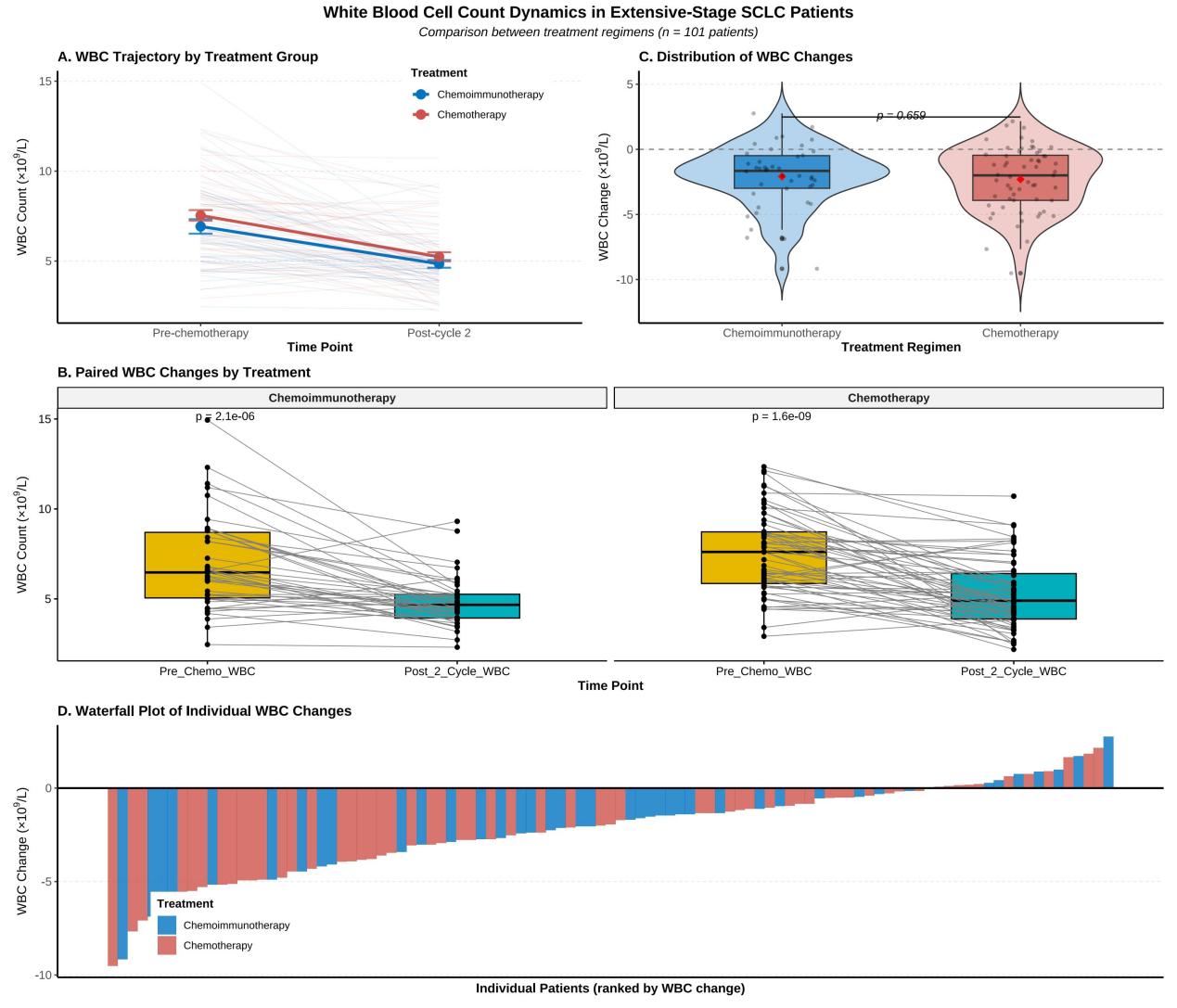
**

**Supplemental Figure 1. WBC Changes by Treatment Regimen in Extensive-Stage SCLC** (A) Mean±SD WBC trajectory from baseline (pre-chemotherapy) to post-cycle 2 by treatment regimen. Chemo-immunotherapy (n = 49); chemotherapy (n = 52). (B) Paired WBC changes from baseline to post-cycle 2 within each treatment arm. Horizontal bars indicate medians; boxes, IQR; whiskers, 1.5 × IQR. The decline was significantly larger in the chemo-immunotherapy group (P = 2.1 × 10^-6^, Wilcoxon signed-rank test). (C) Distribution of absolute (upper panel) and percentage (lower panel) WBC change from baseline to post-cycle 2. Red, chemo-immunotherapy; blue, chemotherapy. (D) Waterfall plot of individual absolute WBC changes (10^9^ cells/L) ranked from largest increase (left) to largest decrease (right). Each bar represents one patient; color denotes treatment regimen as in (C).
